# Knowledge, attitude, and practice toward postoperative self-management among kidney transplant recipients

**DOI:** 10.1186/s12909-024-05631-8

**Published:** 2024-06-11

**Authors:** Xiqian Huang, Beihua Xi, Chengjie Xuan, Yi Bao, Lin Wang, Fei Peng

**Affiliations:** 1 Tongji University School of Medicine, Shanghai, 200092 China; 2 Organ Transplantation Center, Rui Jin Hospital Affiliated to Shanghai Jiao Tong University School of Medicine, Shanghai, 200025 China; 3 Clinical Nutrition Department, Rui Jin Hospital Affiliated to Shanghai Jiao Tong University School of Medicine, Shanghai, 200025 China; 4 Department of Nursing, Shanghai Changzheng Hospital, Naval Medicine University, Shanghai, 200003 China

**Keywords:** Knowledge, Attitude, And practice, Postoperative self-management, Kidney transplant, Questionnaire, China

## Abstract

**Background:**

Patient involvement is crucial to the success of kidney transplants. This study aimed to investigate the knowledge, attitude, and practice (KAP) toward postoperative self-management among kidney transplant recipients.

**Methods:**

A web-based cross-sectional study was conducted in Ruijin Hospital (Shanghai, China) between March 24, 2023, and April 15, 2023 in kidney transplant recipients. A questionnaire was designed to collect data about the characteristics of the participants and their KAP toward postoperative self-management. KAP scores were calculated based on participants' responses, using predefined scoring criteria tailored to evaluate each dimension of KAP effectively.

**Results:**

A total of 483 valid questionnaires were collected, including 189 (39.13%) participants aged between 46 and 60 years. The mean score of knowledge, attitude and practice were 23.44 ± 4.87 (possible range: 0–28), 43.59 ± 2.65 (possible range: 10–50), 52.52 ± 4.64 (possible range: 0–58), respectively. The multivariate analysis showed knowledge scores (OR = 1.15, 95% CI = 1.10–1.20, *p* < 0.001), attitude scores (OR = 1.22, 95% CI = 1.12–1.32, *p* < 0.001) and undergone transplantation within 1 year (OR = 3.92, 95% CI = 1.60–9.63, *p* = 0.003) were independently associated with good practice. Knowledge scores (OR = 1.06, 95% CI = 1.02–1.10, *p* = 0.003), attitude scores (OR = 1.16, 95% CI = 1.08–1.25, *p* < 0.001), aged 16–35 years (OR = 0.38, 95% CI = 0.18–0.78, *p* = 0.009), underwent a single kidney transplant surgery (OR = 3.97, 95% CI = 1.28–12.38, *p* = 0.017) were independently associated with medication adherence.

**Conclusions:**

Kidney transplant recipients had good knowledge, positive attitude and good practice toward postoperative self-management. Implementing personalized education, psychological support, and close monitoring strategies is recommended to optimize postoperative self-management in kidney transplant recipients.

**Supplementary Information:**

The online version contains supplementary material available at 10.1186/s12909-024-05631-8.

## Background

The global incidence of end-stage renal disease (ESRD) is on the rise [1]. Renal transplantation stands as the optimal treatment for ESRD. Since its inaugural procedure in 1954, kidney transplantation has gained worldwide traction [2]. Among ESRD patients, living donor kidney transplantation is the favored option due to superior patient and graft survival rates [3]. This procedure holds prominence in the realm of solid organ transplants and serves as a pivotal intervention for individuals grappling with kidney failure. Despite successful surgical outcomes, ongoing self-management remains imperative to sustain the transplanted kidney's function and the patient's overall well-being [4].

Advancements in surgical techniques and immunosuppressants are boosting post-transplant survival rates while also reducing drug-related side effects. Postoperative self-management in kidney transplantation refers to recipients taking charge of their health post-surgery. This includes following medical instructions, taking immunosuppressants as directed, managing diet and weight, staying active, and attending regular medical check-ups. Self-management is vital to prevent rejection, reduce infections, and extend the life of the transplanted kidney. However, kidney transplant recipients still face significant risks like rejection, cardiovascular issues, and infections. Hence, rigorous postoperative care is necessary, involving strict medication and diet adherence. Kidney transplants often fail due to non-compliance with treatment, negatively impacting recipients' quality of life [5]. It was found that 20–37% of adult patients to be non-compliant, and non-compliance contributes to 50% of acute rejection cases and 15% of organ losses, resulting in short- and long-term physical and economic detriments [6, 7].

The Knowledge, Attitudes, and Practices (KAP) framework plays a pivotal role in unraveling the mechanisms of health education aimed at fostering behavioral changes among patients. This is achieved through the utilization of KAP questionnaires, which delve into an individual's understanding, beliefs, and actions. These KAP surveys offer a valuable tool for assessing the impact of intervention programs. Furthermore, a patient's KAP dimensions are consistently recognized as indispensable factors in effective disease management strategies [8, 9].

A previous study underscored the positive correlation between increased awareness and the presence of children in households with improved adherence to COVID-19 preventive measures among solid organ transplant (SOT) patients, while attitudes displayed a more limited impact [10]. In a separate study, researchers highlighted the moderate levels of kidney transplantation knowledge among both candidates and recipients, emphasizing the necessity for targeted health education interventions that consider demographic factors such as education, age, and fertility status to enhance knowledge within this cohort [11]. Despite the widely recognized importance of self-management after kidney transplantation, the impact of patients' knowledge, attitude, and practice on self-management has not been thoroughly studied. Exploring the knowledge, attitudes, and practices of kidney transplant recipients regarding postoperative self-management offers valuable insights into potential shortcomings and areas of concern, serving as a basis for refining postoperative care protocols.

Therefore, this study aimed to investigate the KAP toward postoperative self-management among kidney transplant recipients.

## Methods

### Study design and participants

This cross-sectional study was conducted at Ruijin Hospital between March 24, 2023, and April 15, 2023 among kidney transplant recipients. The inclusion criteria encompassed patients who underwent kidney transplantation (including graft failure and transplant nephrectomy cases), were on long-term oral immunosuppressive medication, and attended regular follow-up at the hospital's kidney transplant outpatient clinic. Exclusion criteria comprised recipients of multi-organ transplants such as heart, liver, or lung, as well as individuals who didn't provide informed consent.

### Questionnaire and quality control

The questionnaire was developed with references to relevant literature [12–15] and guidelines ("Standardized Follow-Up after Kidney Transplantation (2019 edition) [16]"; "Expert Consensus on Perioperative Accelerated Recovery Management of Kidney Transplantation in China (2018 edition) [17]"). After completing the questionnaire, 34 kidney transplant recipients participated in the pretest. The Cronbach’s α was 0.923. We conducted confirmatory factor analysis simultaneously, showing CFI (comparative fit index) = 0.816 (> 0.8 is good), IFI (incremental fit index) = 0.830 (> 0.8 is good), TLI (Tucker-Lewis index) = 0.816 > 0.8 is good, RMSEA (root mean square error of approximation) = 0.054 (< 0.08 is good), and CMIN/DF (chi-square value/degrees of freedom) = 2.417 (1–3: excellent, 3–5: good).

The final questionnaire encompassed four distinct sections: demographic particulars (including gender, age, education, etc.), the knowledge dimension, the attitude dimension, and the practice dimension. The knowledge dimension encompassed a set of 14 items, each allocated a score of 2 for “Understand”, 1 for “Partially understand”, and 0 for “Do not understand”, thereby establishing a scoring continuum spanning 0 to 28 points. The attitude dimension consisted of 10 questions, evaluated via a five-point Likert scale that ranged from "very positive" (5 points) to "very negative" (1 point), thus offering a scoring spectrum of 10 to 50 points. The practice dimension gauged medication adherence using the Morisky Medication Adherence Questionnaire [18–20], comprising 8 items, and evaluated compliance with postoperative follow-up visits via a five-point Likert scale that ranged from "completely compliant" (5) to "completely non-compliant" (1). The comprehensive score range for the practice dimension spanned 0 to 58 points.

The electronic questionnaires were disseminated through a social media platform in China. The distribution was facilitated using a hyperlink generated through an online questionnaire software platform.

### Sample size

According to the formula for calculating the sample size in cross-sectional surveys $\text{n}={\left(\frac{{Z}_{1-\alpha /2}}{\delta }\right)}^{2}\times p\times \left(1-p\right)$. In the formula, "n" represents the sample size for each group, "α" represents the type I error, which is typically set at 0.05, ${Z}_{1-\alpha /2}=1.96$, δ represents the allowable error, typically set at 0.05, and "*p*" is set at 0.5 (as setting it at 0.5 maximizes the value and ensures a sufficiently large sample size). The calculated sample size "*n*" is 384. Considering an estimated questionnaire response rate of 80%, the final plan is to collect 480 valid questionnaires.

### Statistical analysis

SPSS 22.0 (IBM, Armonk, NY, USA) was used for the analysis. Continuous variables were represented by means and standard deviations (SD). The categorical variables were represented by *n* (%). Continuous variables shall be subject to the normality test first. If they conform to the Normal distribution, the comparison between the two groups shall be subject to the t-test. If they do not conform to the Normal distribution, the comparison between the two groups shall be subject to the Wilcoxon Mann Whitney test; Three or more groups of continuous variables conform to Normal distribution with uniform variance, and ANOVA is used for comparison among multiple groups; If it does not conform to Normal distribution, Kruskal Wallis analysis of variance is adopted. Univariate and multivariate logistic regression analyses were used to identify factors associated with good knowledge, positive attitude and good practice which was defined as the score exceeding mean of the score. Ordered univariate and multivariate logistic regression analyses were used to identify factors associated with medication compliance. Variables with *P* < 0.05 in the univariate analysis were entered into the multivariate analysis, and odds ratios (ORs) and 95% confidence intervals (95%CIs) were calculated. A two-sided *P*-value less than 0.05 was considered to be statistically significant.

## Results

In this study, a total of 513 questionnaires were collected, out of which 30 duplicates and those lacking patients' informed consent were excluded, resulting in 483 valid questionnaires for analysis. Among the participants, 189 (39.13%) were aged between 46 and 60 years, while a substantial majority of 366 (75.78%) reported being married. With respect to residency, 305 (63.15%) participants were situated in urban areas. In terms of educational attainment, 226 (46.79%) participants held either a college or undergraduate degree. Regarding employment status, 195 (40.37%) participants were engaged in full-time employment. An impressive 471 (97.52%) participants had undergone a single kidney transplant surgery, with 348 (72.05%) surpassing the three-year mark since their initial transplant. Notably, 469 (97.10%) participants had availed themselves of education regarding post-kidney transplant precautions. In the context of complications, the data revealed that 304 (62.94%) participants reported an absence of complications, while 60 (12.42%) had experienced postoperative infections. Furthermore, underlying medical conditions were evident, with hypertension prevalent among 303 (62.73%) participants, and hyperlipidemia observed in 78 (16.15%) individuals (Table 1).

**Table 1:** Demographic characteristics

Variables	*N* (%)	Knowledge Score		Attitude Score		Practice Score	
		Mean ± SD	*P*	Mean ± SD	*P*	Mean ± SD	*P*
**Total**	483	23.44 ± 4.87		43.59 ± 2.65		52.52 ± 4.64	
**Gender**			0.005		0.031		0.085
Male	309(63.98)	22.97 ± 5.07		43.39 ± 22.72		52.25 ± 4.81	
Female	174(36.02)	24.28 ± 4.39		43.94 ± 2.49		53.00 ± 4.29	
**Age, years**			0.010		0.288		0.981
16–35	81(16.77)	24.83 ± 4.10		43.09 ± 3.29		52.60 ± 5.18	
36–45	168(34.78)	23.66 ± 5.32		43.64 ± 2.76		52.58 ± 4.61	
46–60	189(39.13)	22.71 ± 4.81		43.76 ± 2.20		52.49 ± 4.47	
≥ 61	45(9.32)	23.22 ± 4.18		43.60 ± 2.66		52.28 ± 4.50	
**Marital Status**			0.163		0.515		0.200
Married	366(75.78)	23.21 ± 5.00		43.66 ± 2.72		52.31 ± 4.69	
Single	78(16.15)	24.31 ± 4.22		43.28 ± 2.47		53.07 ± 4.75	
Divorced/Widowed	39(8.07)	23.90 ± 4.78		43.54 ± 2.36		53.38 ± 3.70	
**Residence**			0.038		0.333		0.388
Rural area	92(19.05)	22.70 ± 5.45		43.63 ± 2.65		52.88 ± 4.75	
Urban area	305(63.15)	23.88 ± 4.59		43.48 ± 2.68		52.57 ± 4.49	
Suburban area	86(17.81)	22.71 ± 5.07		43.95 ± 2.56		51.95 ± 5.01	
**Education**			< 0.001		0.227		0.272
Elementary school or below	12(2.48)	19.25 ± 6.97		42.92 ± 1.16		51.31 ± 8.12	
Junior high school	93(19.25)	22.12 ± 6.00		43.38 ± 2.16		52.60 ± 5.02	
High school/Technical school	137(28.36)	23.57 ± 4.63		43.76 ± 2.74		52.78 ± 4.20	
College/Undergraduate	226(46.79)	24.27 ± 3.92		43.69 ± 2.76		52.55 ± 4.44	
Master’s degree or above	15(3.11)	21.40 ± 6.76		42.33 ± 3.50		50.17 ± 5.08	
**Employment**			0.004		0.634		0.127
Full-time employment	195(40.37)	24.29 ± 4.39		43.78 ± 2.83		52.70 ± 4.75	
Unemployment	58(12.01)	22.88 ± 5.88		43.21 ± 2.36		53.17 ± 4.16	
Retired	108(22.36)	22.10 ± 5.00		43.48 ± 2.42		51.74 ± 4.83	
Self-employed/Freelancer/Part-time	92(19.05)	23.53 ± 4.71		43.68 ± 2.58		52.15 ± 4.58	
Homemaker/housewife	24(4.97)	22.88 ± 5.11		43.08 ± 3.23		53.85 ± 3.35	
Student	6(1.24)	26.33 ± 1.97		43.50 ± 1.64		54.83 ± 4.15	
**Per capita income, China Yuan (CNY)**			0.077		0.040		0.863
≤ 2000	58(12.01)	22.71 ± 6.28		43.00 ± 2.76		52.36 ± 4.65	
2001–5000	130(26.92)	22.78 ± 5.17		43.26 ± 2.59		52.44 ± 4.76	
5001–10000	143(29.61)	23.45 ± 4.60		43.68 ± 2.34		52.34 ± 4.87	
10,001–20000	80(16.56)	24.48 ± 3.51		44.22 ± 2.30		53.03 ± 4.04	
> 20,000	72(14.91)	24.06 ± 4.72		43.78 ± 3.41		52.56 ± 4.67	
**Smoking habit**			0.107		0.445		0.172
Smoker	33(6.83)	22.73 ± 5.65		43.09 ± 2.94		51.10 ± 5.29	
Former smoker	118(24.43)	22.75 ± 4.83		43.75 ± 2.49		52.79 ± 4.28	
Non-Smoker	332(68.74)	23.76 ± 4.79		43.58 ± 2.68		52.57 ± 4.68	
**Health Insurance**			0.187		0.839		0.018
Only social medical insurance	426(88.20)	23.46 ± 4.65		43.56 ± 2.67		52.48 ± 4.57	
Only commercial medical insurance	5(1.04)	23.60 ± 6.43		43.20 ± 3.03		51.70 ± 3.05	
Both social medical and commercial medical insurance	49(10.14)	23.63 ± 6.07		43.90 ± 2.61		53.39 ± 4.42	
No insurance	3(0.62)	17.33 ± 10.26		43.33 ± 0.58		44.92 ± 11.67	
**Number of kidney transplant surgeries**			0.215		0.735		0.432
1	471(97.52)	23.40 ± 4.91		43.60 ± 2.66		52.55 ± 4.64	
2	12(2.48)	25.17 ± 2.92		43.33 ± 2.19		51.48 ± 4.45	
**Time since first kidney transplant surgery, years**			0.255		0.452		0.003
< 1	43(8.90)	24.12 ± 4.49		44.07 ± 2.30		54.44 ± 3.26	
1–3	92(19.05)	23.99 ± 4.88		43.49 ± 2.36		53.15 ± 4.64	
> 3	348(72.05)	23.22 ± 4.91		43.56 ± 2.76		52.12 ± 4.71	
**Post-kidney transplant precaution education**			0.008		< 0.001		< 0.001
Received education	469(97.10)	23.54 ± 4.78		43.69 ± 2.49		52.65 ± 4.46	
Did not receive education	14(2.90)	20.07 ± 6.78		40.14 ± 4.97		48.29 ± 7.88	
**Family Experience with Kidney Transplant**			0.537		0.390		0.635
Yes	11(2.28)	22.55 ± 6.58		42.91 ± 2.66		51.86 ± 6.92	
No	472(97.72)	23.46 ± 4.83		43.61 ± 2.65		52.53 ± 4.58	
**Current complications (Multiple choices)**							
None	304(62.94)	23.89 ± 4.58		43.99 ± 2.42		52.95 ± 4.58	
Postoperative infection	60(12.42)	23.03 ± 5.17		42.90 ± 2.38		52.60 ± 4.04	
Rejection reaction	61(12.63)	23.41 ± 5.65		42.85 ± 3.54		52.26 ± 4.61	
Transplanted kidney bleeding	4(0.83)	25.75 ± 4.50		43.00 ± 2.58		55.00 ± 2.45	
Ureteral stenosis	11(2.28)	20.45 ± 5.45		40.73 ± 5.78		49.73 ± 6.68	
Thromboembolic disease	10(2.07)	22.30 ± 4.97		44.30 ± 1.25		50.80 ± 4.92	
Osteoporosis	53(10.97)	21.40 ± 5.35		42.13 ± 2.37		50.73 ± 5.26	
Other	71(14.70)	23.00 ± 5.24		42.86 ± 3.29		52.23 ± 5.17	
**Underlying medical conditions (Multiple choices)**							
None	135(27.95)	24.51 ± 4.31		43.79 ± 2.39		52.97 ± 4.32	
Hypertension	303(62.73)	22.99 ± 4.96		43.46 ± 2.73		52.38 ± 4.71	
Heart disease	43(8.90)	21.86 ± 6.01		43.00 ± 3.77		52.21 ± 5.37	
Hyperlipidemia	78(16.15)	22.12 ± 5.05		43.26 ± 2.72		51.41 ± 4.85	
Diabetes	48(9.94)	22.54 ± 5.12		42.90 ± 2.73		53.06 ± 4.63	
Neoplastic diseases	10(2.07)	26.60 ± 2.07		44.20 ± 2.62		53.13 ± 4.67	
Other	36(7.45)	21.94 ± 5.76		43.19 ± 3.19		52.31 ± 5.18	

The mean score of knowledge, attitude and practice were 23.44 ± 4.87 (possible range: 0–28), 43.59 ± 2.65 (possible range: 10–50), 52.52 ± 4.64 (possible range: 0–58), respectively. Gender was linked to knowledge scores (*p* = 0.005), with females more likely to have higher knowledge scores. Age also played a role in knowledge scores (*p* = 0.010), with the 16–35 and ≥ 61 age groups more likely to exhibit increased knowledge scores compared to the 46–60 age group. Education (*p* < 0.001) and employment status (*p* = 0.004) were associated with knowledge scores, indicating that those with lower education levels and employed participants were less likely to have high knowledge scores. Per capita income demonstrated significance with attitude scores (*p* = 0.040), highlighting that individuals earning 10,001–20000 China Yuan (about $1,386 to $2,772 United States dollar) were more likely to possess positive attitudes. Health insurance influenced practice scores (*p* = 0.018), with those without insurance less likely to adopt recommended practices. Education on post-kidney transplant precautions associated with higher knowledge (*p* = 0.008), attitude (*p* < 0.001), and practice (*p* < 0.001) scores, emphasizing that educated individuals were more likely to excel in all three dimensions (Table 1).

The three knowledge items with the highest understanding rates were as follows: "Post-kidney transplant recipients are not recommended to use medications or foods that claim to enhance the immune system" (K7) with a correctness rate of 83.44%, "Post-kidney transplant, early mobilization is essential, with daily activity goals set to actively prevent deep vein thrombosis" (K8) with a correctness rate of 77.64%, and "Post-kidney transplant recipients should adhere to regular follow-up and routine examinations. In case of any discomfort, especially fever or oliguria, timely medical consultation is necessary" (K14) with a correctness rate of 94.41%. The three items with the lowest correctness rates were "Post-kidney transplant recipients are prone to complications such as pleural effusion, atelectasis, and lung infections. Early respiratory function exercises are effective methods to increase respiratory muscle strength, promote lung expansion, and reduce postoperative complications" (K9) with a correctness rate of 37.47%, "Respiratory function exercise methods include diaphragmatic breathing, lip-pursued breathing, and using a respiratory training device" (K10) with a correctness rate of 41.41%, and "Post-transplant bone diseases are relatively common, with osteoporosis being a prominent condition. Supplementing vitamin D has a beneficial effect on bone mineral density in adult kidney transplant recipients" (K5) with a correctness rate of 59.01% (Supplementary Table 1).

A significant majority of the patients (97.10%) strongly agreed or agreed that kidney transplantation had brought them a new lease on life, invoking gratitude and cherishment (A1). Similarly, a substantial proportion (91.51%) expressed their belief in the importance of following medical advice and dietary management after kidney transplantation (A5). Furthermore, an overwhelming number of patients (96.69%) affirmed their understanding of the significance of adhering to immunosuppressive medications and monitoring drug concentrations in their blood (A6). On a related note, a substantial majority (91.72%) acknowledged the importance of maintaining good mental health in the context of post-kidney transplant self-management (A7). It is noteworthy, however, that a notable segment of patients (38.92%) reported feeling substantial pressure due to the potential for rejection reactions and complications following transplantation (A2) (Supplementary Table 2).

The examination of patients' behaviors within their treatment regimen revealed that in relation to medication-related practices, 43.06% occasionally forgot to adhere to their prescribed medication schedule (Item 1), 7.66% reported instances of missing medication days in the preceding two weeks (Item 2), and 13.87% acknowledged instances of reducing or discontinuing their medication when confronted with a deterioration of symptoms (Item 3). Notably, a substantial 82.40% of patients (P1) affirmed their commitment to following the doctor's advice for regular follow-up visits. Additionally, an impressive 89.65% (P2) promptly sought medical attention in case of discomfort. Furthermore, patients reported adherence to dietary and lifestyle recommendations, with percentages ranging from 61.08% (P3) to 76.81% (P4). Patients also demonstrated their dedication to psychological well-being and self-care practices, with up to 68.74% (P10) prioritizing maintaining a calm mindset (Supplementary Table 3). The results of the binary classification analysis for each dimension are presented in Supplementary Table 4.

The multivariate analysis showed that females (OR = 2.06, 95% CI = 1.25–3.39, *p* = 0.005) and retired (OR = 0.35, 95% CI = 0.18–0.69, *p* = 0.002) were independently associated with good knowledge (> 23.44) (Table 2). The multivariate analysis showed that knowledge scores (OR = 1.05, 95% CI = 1.01–1.09, *p* = 0.016), females (OR = 1.63, 95% CI = 1.08–2.46, *p* = 0.020), unemployed (OR = 0.52, 95% CI = 0.28–0.98, *p* = 0.041), full-time homemakers or housewives (OR = 0.38, 95% CI = 0.17–0.87, *p* = 0.021), and did not receive education on post-kidney transplant precautions (OR = 0.09, 95% CI = 0.01–0.69, *p* = 0.021) were independently associated with positive attitude (> 43.59) (Table 3). The multivariate analysis showed that knowledge scores (OR = 1.15, 95% CI = 1.10–1.20, *p* < 0.001), attitude scores (OR = 1.22, 95% CI = 1.12–1.32, *p* < 0.001) and undergone transplantation within 1 year (OR = 3.92, 95% CI = 1.60–9.63, *p* = 0.003) were independently associated with good practice (> 52.52) (Table 4).

**Table 2:** Univariate and multivariate analysis for good knowledge (> 23.44)

Variables	Univariate Analysis			Multivariate Analysis		
	OR	95%CI	*P*	OR	95%CI	*P*
**Gender**						
Male	Ref			Ref		
Female	1.72	1.17, 2.54	0.006	2.06	1.25, 3.39	0.005
**Age, years**						
16–35	Ref			Ref		
36–45	0.74	0.42, 1.31	0.298	0.74	0.40, 1.39	0.355
46–60	0.46	0.27, 0.81	0.007	0.64	0.33, 1.24	0.186
≥ 61	0.40	0.19, 0.86	0.018	1.00	0.36, 2.79	0.995
**Marital Status**						
Married	Ref			Ref		
Single	1.73	1.02, 2.92	0.040	1.14	0.62, 2.09	0.669
Divorced/Widowed	1.23	0.62, 2.42	0.551	0.99	0.48, 2.05	0.986
**Residence**						
Rural area	0.67	0.42, 1.07	0.096	0.66	0.37, 1.17	0.152
Urban area	Ref			Ref		
Suburban area	0.62	0.38, 1.00	0.049	0.62	0.37, 1.06	0.081
**Education**						
Elementary school or below	0.39	0.12, 1.27	0.119	0.65	0.18, 2.33	0.513
Junior high school	0.56	0.34, 0.91	0.020	0.79	0.43, 1.44	0.438
High school/Technical school	0.77	0.50, 1.19	0.237	1.00	0.62, 1.61	0.990
College/Undergraduate	Ref			Ref		
Master’s degree or above	0.48	0.17, 1.37	0.170	0.36	0.12, 1.10	0.072
**Employment**						
Full-time employment	Ref			Ref		
Unemployment	0.80	0.44, 1.46	0.460	1.00	0.50, 2.00	0.995
Retired	0.40	0.25, 0.65	< 0.001	0.35	0.18, 0.69	0.002
Self-employed/Freelancer/Part-time	0.78	0.47, 1.30	0.336	0.93	0.53, 1.64	0.805
Homemaker/housewife	1.05	0.46, 2.36	0.912	0.77	0.31, 1.88	0.566
Student						
** Per Capita Income, CNY**	1.24	0.66, 2.32	0.508			
≤ 2000	0.70	0.44, 1.13	0.148			
2001–5000	Ref					
5001–10000	1.24	0.70, 2.17	0.463			
10,001–20000	1.40	0.78, 2.54	0.261			
**Smoking habit**						
Smoker	0.72	0.35, 1.49	0.381	0.99	0.45, 2.16	0.973
Former smoker	0.62	0.41, 0.95	0.030	0.83	0.51, 1.37	0.469
Non-Smoker	Ref			Ref		
**Health Insurance**						
Only social medical insurance	2.76	0.25, 30.67	0.409			
Only commercial medical insurance	3.00	0.15, 59.89	0.472			
Both social medical and commercial medical insurance	4.53	0.38, 53.93	0.232			
No insurance	Ref					
**Number of kidney transplant surgeries**						
1	Ref					
2	2.12	0.57, 7.93	0.264			
**Time since first kidney transplant surgery, years**						
< 1	1.79	0.90, 3.55	0.096			
1–3	1.39	0.86, 2.23	0.178			
> 3	Ref					
**Post-kidney transplant precaution education**						
Received education	Ref					
Did not receive education	0.38	0.12, 1.14	0.083			
**Family Experience with Kidney Transplant**						
Yes	Ref					
No	1.20	0.36, 4.00	0.761

**Table 3:** Univariate and multivariate analysis for positive attitude (> 43.59)

Variables	Univariate Analysis			Multivariate Analysis		
	OR	95%CI	*P*	OR	95%CI	*P*
**Knowledge score**	1.07	1.03, 1.11	0.001	1.05	1.01, 1.09	0.016
**Gender**						
Male	Ref			Ref		
Female	1.55	1.07, 2.26	0.021	1.63	1.08, 2.46	0.020
**Age, years**						
16–35	Ref					
36–45	1.25	0.73, 2.13	0.411			
46–60	1.35	0.80, 2.27	0.265			
≥ 61	1.31	0.63, 2.71	0.473			
**Marital Status**						
Married	Ref					
Single	0.85	0.52, 1.38	0.500			
Divorced/Widowed	0.59	0.30, 1.15	0.121			
**Residence**						
Rural area	0.97	0.61, 1.55	0.906			
Urban area	Ref					
Suburban area	1.40	0.87, 2.28	0.167			
**Education**						
Elementary school or below	0.44	0.13, 1.51	0.192			
Junior high school	0.73	0.45, 1.18	0.198			
High school/Technical school	0.90	0.59, 1.37	0.614			
College/Undergraduate	Ref					
Master’s degree or above	0.59	0.20, 1.71	0.330			
**Employment**						
Full-time employment	Ref			Ref		
Unemployment	0.46	0.25, 0.84	0.011	0.52	0.28, 0.98	0.041
Retired	0.78	0.48, 1.24	0.292	0.82	0.50, 1.34	0.425
Self-employed/Freelancer/Part-time	0.88	0.53, 1.44	0.610	1.02	0.61, 1.71	0.930
Homemaker/housewife	0.47	0.21, 1.03	0.060	0.38	0.17, 0.87	0.021
Student						
** Per Capita Income, CNY**	0.60	0.32, 1.12	0.111			
≤ 2000	0.72	0.45, 1.17	0.184			
2001–5000	10.56	0.89, 2.72	0.118			
5001–10000	10.46	0.82, 2.60	0.194			
10,001–20000						
**Smoking habit**						
Smoker	0.75	0.36, 1.54	0.427			
Former smoker	1.12	0.74, 1.71	0.596			
Non-Smoker	Ref					
**Health Insurance**						
Only social medical insurance	2.00	0.18, 22.22	0.573			
Only commercial medical insurance	1.33	0.07, 26.62	0.851			
Both social medical and commercial medical insurance	2.08	0.18, 24.51	0.559			
No insurance	Ref					
**Number of kidney transplant surgeries**						
1	Ref					
2	1.00	0.32, 3.16	0.994			
**Time since first kidney transplant surgery, years**						
< 1	1.25	0.66, 2.36	0.495			
1–3	0.83	0.52, 1.32	0.429			
> 3	Ref					
**Post-kidney transplant precaution education**						
Received education	Ref			Ref		
Did not receive education	0.07	0.01, 0.57	0.012	0.09	0.01, 0.69	0.021
**Family Experience with Kidney Transplant**						
Yes	Ref					
No	1.20	0.36, 3.99	0.766

**Table 4:** Univariate and multivariate analysis for good practice (> 52.52)

Variables	Univariate Analysis			Multivariate Analysis		
	OR	95%CI	*P*	OR	95%CI	*P*
**Knowledge score**	1.17	1.12, 1.23	< 0.001	1.15	1.10	< 0.001
**Attitude score**	1.28	1.17, 1.39	< 0.001	1.22	1.12	< 0.001
**Gender**						
Male	Ref					
Female	1.37	0.94, 2.00	0.106			
**Age, years**						
16–35	Ref					
36–45	0.89	0.51, 1.53	0.666			
46–60	0.74	0.43, 1.25	0.260			
≥ 61	0.74	0.35, 1.54	0.416			
**Marital Status**						
Married	Ref					
Single	1.59	0.95, 2.66	0.078			
Divorced/Widowed	1.59	0.79, 3.19	0.193			
**Residence**						
Rural area	1.36	0.83, 2.20	0.219			
Urban area	Ref					
Suburban area	0.79	0.49, 1.28	0.346			
**Education**						
Elementary school or below	0.98	0.30, 3.18	0.972			
Junior high school	0.93	0.57, 1.51	0.759			
High school/Technical school	1.07	0.70, 1.66	0.744			
College/Undergraduate	Ref					
Master’s degree or above	0.47	0.16, 1.35	0.161			
**Employment**						
Full-time employment	Ref			Ref		
Unemployment	1.05	0.57, 1.94	0.865	1.42	0.71, 2.84	0.319
Retired	0.54	0.33, 0.86	0.010	0.80	0.47, 1.36	0.407
Self-employed/Freelancer/Part-time	0.74	0.45, 1.23	0.250	0.82	0.47, 1.43	0.484
Homemaker/housewife	1.40	0.61, 3.21	0.432	2.03	0.79, 5.23	0.141
Student						
**Per Capita Income, CNY**	1.08	0.58, 2.01	0.797			
≤ 2000	0.92	0.57, 1.49	0.736			
2001–5000	Ref					
5001–10000	1.42	0.81, 2.50	0.223			
10,001–20000	1.20	0.67, 2.14	0.531			
**Smoking habit**						
Smoker	0.52	0.25, 1.07	0.075			
Former smoker	1.14	0.74, 1.75	0.552			
Non-Smoker	Ref					
**Health Insurance**						
Only social medical insurance	2.66	0.24, 29.51	0.427			
Only commercial medical insurance	1.33	0.07, 26.62	0.851			
Both social medical and commercial medical insurance	5.54	0.46, 66.32	0.177			
No insurance	Ref					
**Number of kidney transplant surgeries**						
1	Ref					
2	0.71	0.22, 2.22	0.553			
**Time since first kidney transplant surgery, years**						
< 1	4.28	1.85, 9.87	0.001	3.92	1.60, 9.63	0.003
1–3	1.29	0.81, 2.07	0.282	1.18	0.70, 1.98	0.534
> 3	Ref			Ref		
**Post-kidney transplant precaution education**						
Received education	Ref					
Did not receive education	0.52	0.18, 1.54	0.239			
**Family Experience with Kidney Transplant**						
Yes	Ref					
No	0.52	0.14, 1.98	0.337			

**Fig. 1:**
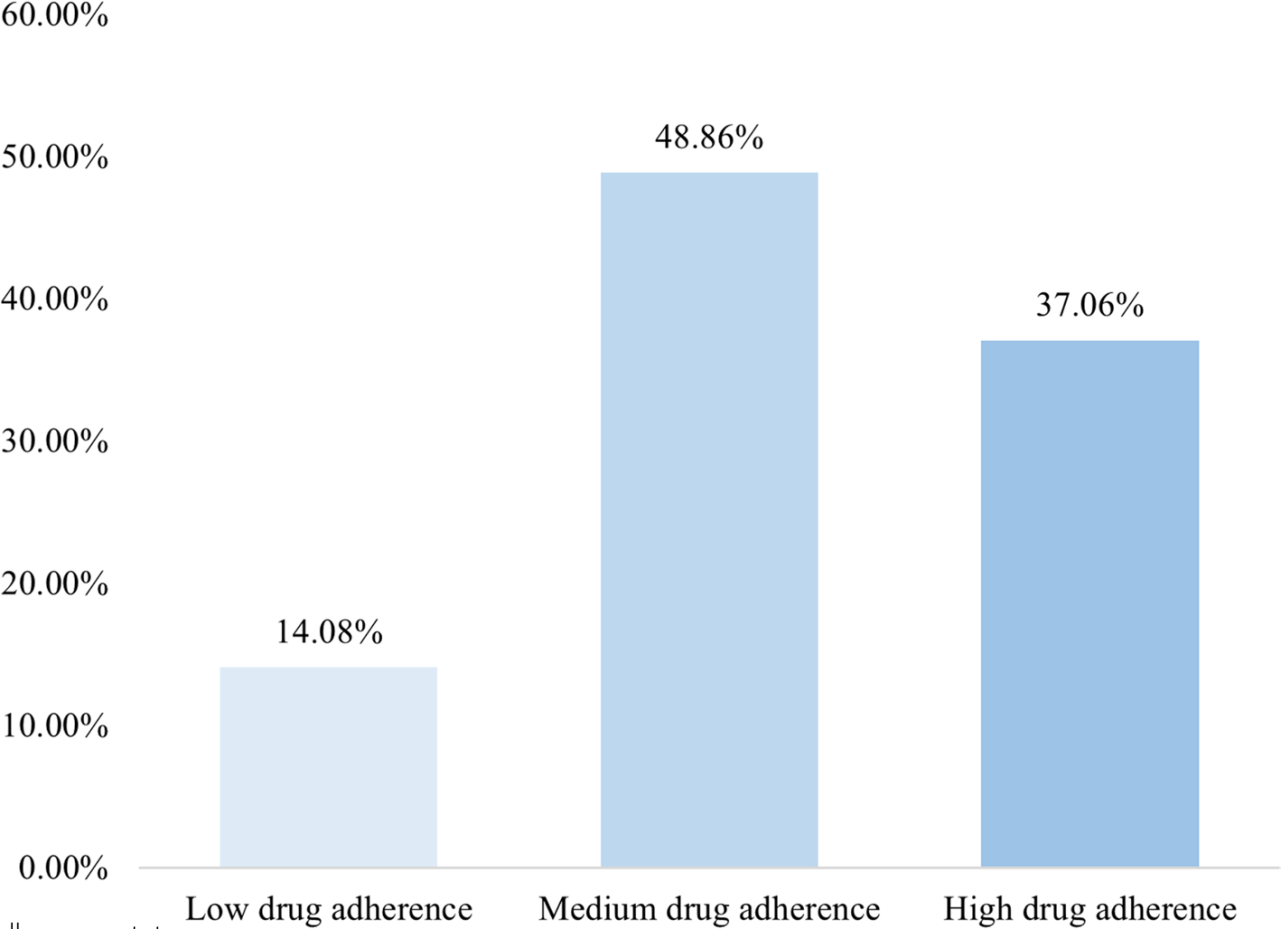
Medication adherence status

The multivariate analysis showed that knowledge scores (OR = 1.06, 95% CI = 1.02–1.10, *p* = 0.003), attitude scores (OR = 1.16, 95% CI = 1.08–1.25, *p* < 0.001), aged 16–35 years (OR = 0.38, 95% CI = 0.18–0.78, *p* = 0.009), underwent a single kidney transplant surgery (OR = 3.97, 95% CI = 1.28–12.38, *p* = 0.017) were independently associated with medication adherence (Table 5). Medication Adherence Status is shown in Fig. 1.

**Table 5:** Univariate and multivariate analysis for medication adherence

Variables	Univariate Analysis			Multivariate Analysis		
	OR	95%CI	*P*	OR	95%CI	*P*
**Knowledge score**	1.07	1.03,1.11	< 0.001	1.06	1.02, 1.10	0.003
**Attitude score**	1.20	1.12,1.29	< 0.001	1.16	1.08, 1.25	< 0.001
**Gender**						
Male	0.82	0.58,1.17	0.274			
Female	Ref					
**Age, years**						
16–35	0.47	0.23,0.94	0.032	0.38	0.18, 0.78	0.009
36–45	0.75	0.40,1.40	0.366	0.62	0.32, 1.19	0.149
46–60	0.98	0.52,1.81	0.936	0.84	0.44, 1.59	0.587
≥ 61	Ref			Ref		
**Marital Status**						
Married	1.43	0.76, 2.69	0.264			
Single	1.63	0.78, 3.39	0.191			
Divorced/Widowed						
**Residence**						
Rural	0.98	0.56, 1.72	0.952			
Urban	1.10	0.69, 1.73	0.694			
Suburban	**Ref**					
**Education**						
Elementary school or below	0.87	0.21, 3.71	0.518			
Junior high school	1.10	0.39, 3.11	0.856			
High school/Technical school	1.37	0.50,3.78	0.855			
College/Undergraduate	1.39	0.51,3.75	0.543			
Master’s degree or above	Ref					
**Employment**						
Employed (full time)	0.89	0.43, 1.84	0.748			
Unemployed	0.85	0.37, 1.97	0.705			
Retired	0.91	0.42, 1.96	0.802			
Self-employed/Freelancer/Part-time	0.62	0.28, 1.35	0.227			
Full-time homemaker/housewife	Ref					
Student						
** Per Capita Income, CNY**	0.83	0.49, 1.43	0.507			
≤ 2000	0.55	0.28, 1.06	0.076			
2001–5000	0.76	0.44, 1.31	0.316			
5001–10000	0.93	0.51, 1.71	0.821			
10,001–20000	Ref					
**Smoking habit**						
Yes	1.00	0.51, 1.97	0.998			
Former smoker	0.87	0.58, 1.30	0.496			
No						
**Health Insurance**						
Only social medical insurance (e.g., employee medical insurance, “New Rural Cooperative Medical System,” “Urban Resident Basic Medical Insurance)	0.52	0.06, 4.80	0.566			
Only commercial medical insurance	0.42	0.03, 6.75	0.543			
Both social medical insurance and commercial medical insurance	0.36	0.04, 3.51	0.380			
No insurance	Ref					
**Times of kidney transplant surgeries**						
1	3.12	1.04, 9.34	0.042	3.97	1.28, 12.38	0.017
2	Ref			Ref		
**For your first kidney transplant, how long has it been since the surgery**						
< 1 year	1.74	0.94, 3.20	0.076			
1–3 years	1.45	0.93, 2.24	0.100			
3 years or more	Ref					
**Education on post-kidney transplant precautions**						
Yes	2.88	1.04, 7.96	0.041	1.48	0.50, 4.38	0.474
No	Ref					
**Do other members of your family have experience with kidney**						
Yes	0.41	0.13, 1.29	0.128			
No	Ref

## Discussion

Kidney transplant recipients exhibited good knowledge, positive attitude and good practice toward postoperative self-management. To enhance the efficacy of postoperative self-management among kidney transplant recipients, it is advisable to integrate tailored educational interventions, psychological support mechanisms, and vigilant monitoring strategies.

The assessment of knowledge, attitude, and practice scores provides a comprehensive understanding of patients' management of post-kidney transplant care. While reasonable mean scores for knowledge and positive attitude signify foundational awareness and motivation to adhere to care recommendations, there exists room for improvement in the practice domain. Although patients possess knowledge and positive attitudes, effective translation into consistent actions necessitates targeted interventions. These findings are consistent with similar studies on chronic disease management and self-care [21, 22]. The observed correlation between higher education and improved knowledge scores supports existing research highlighting education's role in health literacy [23–25].

The demographic distribution of participants in this study reflects trends observed in previous research on kidney transplant recipients. Studies consistently show that the majority of transplant recipients fall within the age range of 46 to 60 years, likely due to the higher incidence of kidney diseases in older adults [26, 27]. The prevalence of married participants aligns with research indicating that social support, often provided by spouses, positively impacts post-transplant outcomes [28, 29]. Similarly, the higher educational attainment observed here corresponds with literature highlighting education's influence on health behaviors and adherence [30]. This demographic profile offers insights into the population under investigation, where participants' predominant age range of 46 to 60 years and their married status could reflect the prevalence of kidney disease and transplantation among middle-aged individuals. The concentration of participants in urban areas might indicate better access to healthcare facilities and transplant centers. Moreover, the prevalence of higher educational attainment suggests that educational background could play a role in patients' understanding and engagement in post-transplant care.

The variability in the accuracy of knowledge items emphasizes the significance of targeted educational interventions, aligning with analogous findings in studies concentrating on patient education for chronic disease management [31]. Notably, challenges in comprehending certain items, including respiratory exercises and post-transplant bone health, resonate with the intricate nature of medical information, potentially necessitating simplified and patient-centric explanations [32]. The identification of individual knowledge items with varying correctness rates serves to highlight sectors warranting focused patient education endeavors. Items with elevated correctness rates denote effective communication of general post-transplant precautions. In contrast, the struggles in grasping specific elements like respiratory exercises and post-transplant bone health accentuate opportunities for fortifying patient education in these domains. By addressing these gaps through tailored educational strategies, a more comprehensive level of patient knowledge can be achieved.

The participants' favorable attitudes towards kidney transplantation and their adherence to medical advice align with the recognized positive influence of transplantation on patients' quality of life [33, 34]. Correspondingly, the acknowledgment of pressure stemming from potential complications resonates with the psychological burden commonly faced by transplant recipients, as indicated by qualitative research [35, 36]. The positive attitudes exhibited by patients towards kidney transplantation and their dedication to medical guidance and dietary management signify their recognition of the procedure's benefits, playing a pivotal role in effective post-transplant care. Nevertheless, a notable subset of patients encountering pressure due to potential complications accentuates the psychological challenges associated with transplantation. Introducing psychological support into the care regimen stands as a potential strategy to alleviate these concerns and foster overall well-being.

The documented cases of medication non-adherence, encompassing instances of forgetting medication schedules, underscore well-established difficulties encountered in managing chronic diseases [37]. Additionally, the notable dedication to follow-up appointments and timely medical attention mirrors findings that underscore the pivotal role of healthcare provider-patient relationships in enhancing patient engagement [38]. Through an examination of medication-related behaviors, insights into challenges pertaining to adhering to prescribed regimens emerge. The occurrences of missed medication schedules and doses illuminate specific areas necessitating interventions aimed at bolstering adherence. Moreover, the elevated rates of commitment demonstrated in follow-up visits and the prompt pursuit of medical care serve as encouraging indications of robust patient engagement. Notably, the adherence to dietary and lifestyle recommendations further underscores patients' active involvement in their care.

The pertinence of gender, age, retirement status, education, and employment in shaping knowledge, attitude, practice, and medication adherence aligns cohesively with existing research that underscores socio-demographic factors as pivotal predictors of health-related behaviors [39]. The multivariate analyses conducted in this study provide a thorough understanding of the intricate factors that exert influence over various dimensions of patient care. These insightful findings subsequently facilitate the customization of interventions based on the unique characteristics of individual patients. The observed impact of gender, age, retirement status, educational level, and employment status across knowledge, attitude, practice, and medication adherence further underscores the imperative of addressing a diverse array of patient needs.

This study had several limitations. Firstly, the cross-sectional design restricts our ability to infer causality or establish temporal relationships between variables. Secondly, the reliance on self-reported data might introduce recall bias or social desirability bias, potentially compromising the accuracy of responses. Thirdly, the study's focus on a single hospital may limit the generalizability of the findings to broader populations. Moreover, the use of an online survey could exclude individuals without internet access or those less comfortable with digital interfaces, potentially omitting significant demographic groups. Importantly, the underrepresentation of younger adults, particularly those aged 20 and below, in our study population suggests a need for caution in extrapolating our findings to this subgroup.

## Conclusions

Kidney transplant recipients had good knowledge, positive attitude and good practice toward postoperative self-management. Targeted educational modules to address knowledge gaps, integrated psychosocial support, adherence strategies, and patient engagement are recommended. Emphasizing follow-up, lifestyle compliance, adopting a holistic care approach with specialists, and establishing ongoing assessment mechanisms are also recommended.

### Supplementary Information

Supplementary Material 1.

## Data Availability

All data generated or analysed during this study are included in this published article and its supplementary information files.

## Abbreviations

KAP: Knowledge, attitude, and practice
ESRD: End-stage renal disease
SOT: Solid organ transplant
CNY: China Yuan
