# Supplementary material for: Knowledge, attitude, and practice toward postoperative self-management among kidney transplant recipients
Source: BMC Med Educ. 2024 Jun 11;24:652. doi: 10.1186/s12909-024-05631-8 (PMC11167737; doi:10.1186/s12909-024-05631-8)
Supplement: Supplementary file 1 — Supplementary Material 1. [file 12909_2024_5631_MOESM1_ESM.doc]

**Supplementary Table 1** Responses to Each Item of Knowledge.

|  | **Understand n (%)** | **Partially understand n (%)** | **Do not understand n (%)** |
| --- | --- | --- | --- |
| K1: Post-transplant malnutrition stands as a key factor impacting recipient recovery, potentially compromising immune response, inciting infections, and delaying wound healing. Conversely, excessive nutrition leading to obesity can strain kidney metabolism and hinder function recuperation. | 359 (74.33) | 116 (24.02) | 8 (1.66) |
| K2: Post-kidney transplant patients should adopt a high-quality protein diet and adapt protein intake according to glomerular filtration rate shifts. | 289 (59.83) | 165 (34.16) | 29 (6.00) |
| K3: For post-kidney transplant recipients, steering clear of sugary drinks and high-sugar diets while maintaining a healthy weight is advised. Blood sugar targets encompass fasting levels of 4-7 mmol/L and pre-meal/nighttime levels of 4-10 mmol/L. | 365 (75.57) | 100 (20.70) | 18 (3.73) |
| K4: Vigilance towards a low-fat diet and restricting high-cholesterol foods is essential for post-kidney transplant recipients. | 372 (77.02) | 103 (21.33) | 8 (1.66) |
| K5: Common post-transplant bone ailments, particularly osteoporosis, highlight the value of vitamin D supplementation in bolstering bone mineral density among adult kidney transplant recipients. | 285 (59.01) | 149 (30.85) | 49 (10.14) |
| K6: Given the propensity for post-kidney transplant complications like hypertension, hypocalcemia, hyperphosphatemia, and hyperkalemia, meticulous control over sodium and potassium intake is necessary. | 322 (66.67) | 135 (27.95) | 26 (5.38) |
| K7: It is inadvisable for post-kidney transplant recipients to resort to medications or foods claiming to enhance the immune system. | 403 (83.44) | 71 (14.70) | 9 (1.86) |
| K8: Promoting early mobility post-kidney transplant is critical, setting daily activity goals to actively prevent deep vein thrombosis. | 375 (77.64) | 90 (18.63) | 18 (3.73) |
| K9: Respiratory issues post-kidney transplant, such as pleural effusion and lung infections, underscore the efficacy of early respiratory function exercises in fortifying respiratory muscle strength and minimizing postoperative complications. | 266 (37.47) | 170 (35.20) | 47 (9.73) |
| K10: Techniques like diaphragmatic breathing, lip-pursued breathing, and respiratory training devices are recommended for respiratory function exercises. | 181 (37.47) | 200 (41.41) | 102 (21.12) |
| K11: After a kidney transplant, maintaining regular bowel movements is vital, aiming for 1-2 soft bowel movements per day and promptly seeking medical aid for constipation while avoiding straining during defecation. | 351 (72.67) | 105 (21.74) | 27 (5.59) |
| K12: Emphasizing infection prevention post-kidney transplant is paramount. Prudent measures include avoiding overexertion, crowded environments, and ensuring indoor ventilation for reduced infection risks. | 443 (91.72) | 35 (5.59) | 5 (1.04) |
| K13: Post-kidney transplant patients must diligently adhere to prescribed immunosuppressive drug regimens. Notably, drugs like cyclosporine, tacrolimus, and sirolimus should not be ingested alongside grapefruit or grapefruit juice. | 450 (93.17) | 27 (5.59) | 6 (1.24) |
| K14: Sustaining regular follow-up and routine examinations is crucial for post-kidney transplant recipients. In the event of discomfort, particularly fever or reduced urine output, timely medical consultation is imperative. | 456 (94.41) | 23 (4.76) | 4 (0.83) |

**Supplementary Table 2 Responses to Each Item of Attitude.**

|  | **Strongly Agree** | **Agree** | **Neutral** | **Disagree** | **Strongly Disagree** | **Score, Mean ± SD** |
| --- | --- | --- | --- | --- | --- | --- |
| A1: Kidney transplantation has bestowed upon you a renewed lease on life, evoking profound gratitude and a commitment to treasuring this opportunity. | 469 (97.10) | 10 (2.07) | 2 (0.41) | 1 (0.21) | 1 (0.21) | 1.04±0.30 |
| A2: The potential for rejection reactions and other post-transplant complications brings about a significant burden of stress and concern. | 188 (38.92) | 111 (22.98) | 135 (27.95) | 28 (5.80) | 21 (4.35) | 2.14±1.13 |
| A3: Your conviction in the vastly improved quality of life post-kidney transplantation compared to your experiences with hemodialysis or peritoneal dialysis is unwavering. | 454 (94.00) | 26 (5.38) | 3 (0.62) | 0 | 0 | 1.07±0.27 |
| A4: Firmly believing in the extension of the transplanted kidney's lifespan through diligent adherence to scheduled outpatient follow-ups, in alignment with medical guidance, holds long-term survival significance. | 460 (95.24) | 21 (4.35) | 1 (0.21) | 0 | 1 (0.21) | 1.06±0.29 |
| A5: The paramount importance of heeding medical advice and meticulously managing your diet subsequent to kidney transplantation resonates strongly with you. | 442 (91.51) | 37 (7.66) | 1 (0.21) | 0 | 0 | 1.10±0.36 |
| A6: Your comprehension of the pivotal role in taking immunosuppressive medications as per medical instructions, consistently monitoring drug concentration in your bloodstream, and making dosage adjustments when necessary is steadfast. | 467 (96.69) | 13 (2.69) | 2 (0.41) | 1 (0.21) | 0 | 1.04±0.25 |
| A7: Recognizing the integral role of nurturing positive mental well-being, you perceive it as a cornerstone of effective self-management post-kidney transplant. | 443 (91.72) | 36 (7.45) | 2 (0.41) | 1 (0.21) | 1 (0.21) | 1.10±0.37 |
| A8: You perceive substantial value in participating in a patient support group, which provides an avenue for learning pertinent information, gaining support from peers, and accessing insights and strategies from fellow patients to enhance your health management. | 397 (82.19) | 58 (12.01) | 26 (5.38) | 2 (0.41) | 0 | 1.24±0.56 |
| A9: While acknowledging the advantages of a patient support group, you are aware that exposure to fellow patients' negative experiences and complications could potentially introduce additional pressure and anxiety. | 118 (24.43) | 103 (21.33) | 148 (30.64) | 74 (15.32) | 40 (8.28) | 2.62±1.24 |
| A10: Your self-assurance in your ability to manage the disease is a resolute guiding force. | 277 (57.35) | 164 (33.95) | 39 (8.07) | 3 (0.62) | 0 | 1.52±0.67 |

**Supplement table 3 Responses to Each Item of practice.**

|  | **Yes** |  | **No** |  |  | **Score, Mean ± SD** |
| --- | --- | --- | --- | --- | --- | --- |
| **Patients' actions within their treatment regimen** |  |  |  |  |  |  |
| 1. During treatment, do you sometimes forget to take your medication? | 208(43.06) |  | 275(56.94) |  |  | 0.57±0.50 |
| 2. In the past two weeks, was there a day or several days when you forgot to take your medication? | 37(7.66) |  | 446(92.34) |  |  | 0.92±0.27 |
| 3. During treatment, when you feel that your symptoms worsen or experience other symptoms, do you reduce or stop taking your medication without informing your doctor? | 67(13.87) |  | 416(86.13) |  |  | 0.86±0.35 |
| 4. When traveling or being away from home for an extended period, have you ever forgotten to bring your medication with you? | 52(10.77) |  | 431(89.23) |  |  | 0.89±0.31 |
| 5. Did you take your medication on time yesterday? | 462(95.65) |  | 21(4.35) |  |  | 0.96±0.20 |
| 6. When you feel that your condition is under control, have you ever stopped taking your medication without informing your doctor? | 21(4.35) |  | 462(95.65) |  |  | 0.96±0.20 |
| 7. Do you find it difficult to adhere to the treatment plan? | 33(6.83) |  | 450(93.17) |  |  | 0.93±0.25 |
|  | **Never** | **Occasionally** | **Sometimes** | **Frequently** | **Always** |  |
| 8. Do you find it challenging to remember to take your medication on time and in the correct dosage? | 348(72.05) | 113(23.40) | 15(3.11) | 3(0.62) | 4(0.83) | 0.91±0.16 |
| **Compliance with Postoperative Follow-up Visits** | **Strongly Agree** | **Agree** | **Neutral** | **Disagree** | **Strongly Disagree** |  |
| 1. After kidney transplantation, you will follow the doctor's advice for regular follow-up visits. | 398(82.40) | 80(16.56) | 3(0.62) | 1(0.21) | 1(0.21) | 4.81±0.45 |
| 2. After kidney transplantation, if you experience discomfort such as fever or oliguria, you will promptly seek medical attention. | 433(89.65) | 42(8.70) | 6(1.24) | 1(0.21) | 1(0.21) | 4.87±0.42 |
| 3. You will follow the doctor's advice on your diet and nutrition. | 295(61.08) | 173(35.82) | 11(2.28) | 2(0.41) | 2(0.41) | 4.57±0.61 |
| 4. You will proactively avoid foods that can affect drug concentration in your blood. | 371(76.81) | 86(17.81) | 12(2.48) | 3(0.62) | 11(2.28) | 4.66±0.76 |
| 5. You will proactively avoid foods that claim to enhance the immune system. | 342(70.81) | 96(19.88) | 25(5.18) | 13(23.69) | 7(1.45) | 4.56±0.83 |
| 6. You will engage in appropriate rehabilitation exercises, such as respiratory function exercises. | 249(51.55) | 155(32.09) | 59(12.22) | 16(3.31) | 4(0.83) | 4.30±0.87 |
| 7. You will proactively minimize visits to crowded places. | 281(58.18) | 148(30.64) | 41(83.49) | 12(2.48) | 1(0.21) | 4.44±0.77 |
| 8. You will take self-protection measures and regularly disinfect your living environment. | 241(49.90) | 173(35.82) | 49(10.14) | 18(3.73) | 2(0.41) | 4.31±0.83 |
| 9. You will maintain a good daily routine and avoid overexertion. | 245(50.72) | 188(38.92) | 41(8.49) | 6(1.24) | 3(0.62) | 4.38±0.75 |
| 10. You will pay attention to maintaining a calm mindset and a positive psychological state. | 332(68.74) | 121(25.05) | 25(5.18) | 4(0.83) | 1(0.21) | 4.61±0.65 |

**Supplementary Table 4 Binary Classification of Each Dimension.**

|  | N (%) |
| --- | --- |
| Knowledge |  |
| [0, 23.44] | 198 (40.99) |
| (23.44, 28] | 285 (59.01) |
| Attitude |  |
| [10, 43.59] | 242 (50.10) |
| (43.59, 50] | 241 (49.90) |
| Practice |  |
| [10, 52.52] | 201 (41.61) |
| (52.52, 58] | 282 (58.39) |
